# A Qualitative Study on Representations of Intellectual and Relational Capital Among a Group of Managers in an Italian Trade-Union

**DOI:** 10.3389/fpsyg.2021.641584

**Published:** 2021-07-01

**Authors:** Silvio Carlo Ripamonti, Laura Galuppo, Sara Petrilli, Angelo Benozzo

**Affiliations:** ^1^Department of Psychology, Catholic University of Milan, Milan, Italy; ^2^Department of Human and Social Science, University of Valle d'Aosta, Aosta, Italy

**Keywords:** intangible assets, intellectual capital, relational capital, root metaphors, trade union

## Abstract

The way in which managers perceive their organization's intellectual and social capital has an impact in shaping their choices and how they lead change. The aim of the study was to explore how the managers of a trade union framed the role of its intangible assets in a context of organizational change. A qualitative approach was used; 30 semi-structured interviews were conducted with the leaders of a trade union and then analyzed using the method of thematic analysis. Particular attention was paid to the metaphors the managers used to narrate change. The hypothesis underlying this approach is that metaphors are a meaningful resource in that they can convey how organization and its intangible assets are framed. In the results, three “root metaphors” are illustrated—the trade union seen either as a system of domination, an organism, or a culture—together with the consequences of each of these images for the perception and value attributed to the trade union's intangible assets. In conclusion, implications for changing management practices and for further research are discussed.

## Introduction

This article presents a study that explored how the managers of a large Italian trade union organization perceive and give value to their intangible assets—intellectual capital and relational capital—within a context of organizational change. Starting from a socio-constructionist perspective, we assume that the social capital of an organization is both the object and the product of sensemaking and negotiation processes. Drawing on Benevene and her colleagues, we argue that the way in which managers “*perceive their organization's intangible resources has an impact in shaping strategic analysis and choices and ultimately, the organization's management*” (Benevene et al., 2019, p. 163).

This is even more true in situations of organizational crisis, such as those that the union has been experiencing for some years now: “*In a neoliberal political economy the space for independent trade unionism is continually narrowed as the state seeks to release market forces from inhibiting regulation and employers*” (Heery, 2009, p. 327). Today, the situation is made even more complicated by the presence in the world of work of other forms of representation such as company councils and civic activism (community organizations, NGOs), which in many contexts represent the interests of workers (Charlwood and Terry, 2007).

Today, in order to confront this identity crisis, the union and its managers are engaged in a process of re-framing both their social and economic function and their intangible assets. Framing is a powerful mechanism used to support and orient those processes of social mobilization which are needed in order for the union to respond to its changing identity and activate consensus. For the purposes of our study, it is useful to recall the concept of “collective action framing,” that is, that mechanism of construction of meanings and beliefs which “*inspire and legitimate the activities and campaigns of a social movement organization*” (Benford and Snow, 2000, p. 614). Framing performs the function of activating consensus and mobilizing action through (a) identifying and defining a problem (diagnostic framing); (b) defining the responsibilities arising from that problem; (c) defining solutions (prognostic framing) and (d) motivating action (motivational framing).

Currently, the union management is developing various new types of “collective action framing,” which differ from each other in terms of how the situation is defined (why are we in a crisis?); how the purpose is identified (what new purpose should the union have?) and the type of action necessary to achieve its goals (what tools and resources can be mobilized to obtain consensus and get action?).

Within these collective frameworks, a central role is played by the intangible assets which the union has available to “mobilize” its action. In particular, it seems to us that that the assets most intimately involved are:

- *human capital*- understood as a set of knowledge, skills, and talents present in the organization, which may be activated or renewed in the ongoing change (Mubarik et al., 2018);
- *relational capital -* understood as the relational assets that characterize the relationship with its own stakeholders (Kong, 2010); in the organization we are dealing with, this refers to the process of representation, that is, the different kind of relationship that the union has with its members (workers), with other potential target users of their services, and with employers and the state (the traditional counterparts in the negotiating process).

The research illustrated below highlights how, when faced with the loss of social prestige and the questioning of the very reasons for the existence of the trade union organization, the managers use processes of reframing the union's actions in order to implement new ways of mobilizing the workers. These processes involve the ability to see, imagine, and give value to the union's intangible assets.

The framing process has been explored in many studies, using various methodological approaches. The hypothesis proposed here is that imaginative thinking is a resource that supports the sensemaking and framing process, and that it is a particularly powerful way of mobilizing the audience. Within this perspective, the use of metaphors and other linguistic devices is especially important (Barley, 1983; Tsouskas, 1991; Cassell and Lee, 2012; Morgan, 2016; Gherardi et al., 2017).

The present study sets out to investigate the process by which organizational change is framed within a trade union, and also the role played by the union's intangible assets, by analyzing the metaphors used by some trade union managers. In the context of the present-day crisis of legitimacy experienced by trade unions (Ripamonti et al., 2021), this study is innovative and bridges a gap both with regard to the use of metaphors to understand the framing process, and with regard to the topic of intangible assets.

## The Research

The research described here is part of a wider study (Ripamonti et al., 2021) focused on a process of organizational change—involving cost-cutting measures—which regarded the merging of two regional branches in northern Italy belonging to one of the most important trade unions in the country. The union in question contacted the researchers and asked for assistance during the process of change. The research protocol consisted of conducting 30 qualitative interviews with the leaders of the trade union association.

The participants are union leaders who were directly involved in a process aimed at reorganizing two important local federations in Northern Italy. They all have managerial responsibility for work groups but also a role in the steering group at the regional and national level. They are therefore in a position to exert influence on how the guidelines of the national trade union organization are interpreted locally. In the course of the interviews, we asked them to tell us which paths of development they thought the union should focus upon in order to reinforce its capacity to influence the labor market and enhance its image in the eyes of its members.

The interviews lasted about an hour and were conducted by one of the authors of the paper. Each interview was recorded and then fully transcribed and anonymized. The data-set was then analyzed using the thematic analysis technique, which made it possible to identify the metaphors described in this article.

One part of the study consisted of analyzing the metaphors which the managers used in order to narrate the changes taking place in the organization. The underlying idea was that by interpreting the metaphors used, it might be possible to understand which concepts of organization are present among the organizational actors and also what the intangible assets of this trade union organization might be, together with the consequences of this as regards ideas of representation.

From a methodological point of view, the tool used to make these representations emerge was the evocation of metaphors: these can activate a kind of imaginative thought which can serve to find out what kinds of framing are being used to attribute meaning to an organizational context (Velasco et al., 2013; Morgan, 2016; Schoeneborn et al., 2016; Scaratti et al., 2017; D'Angelo et al., 2018; Gozzoli et al., 2018; Taylor and Fairchild, 2020).

In line with a long and well-established tradition in the literature on organization studies (Brown, 1976; Morgan, 1980; Barley, 1983; Tsouskas, 1991; Cassell and Lee, 2012; Gherardi et al., 2017; Benozzo and Gherardi, 2019), metaphors are conceived of not as a rhetorical device used in a text or speech but as a way of generating new knowledge and/or as a heuristic for understanding a specific organization and its complexity, and how its intangible assets are constructed by organizational actors.

We took inspiration from a tradition present in organization studies which dates back to Morgan's (1986) *Images of Organization*: this work unveiled the possibility of using metaphors both for analysis and to intervene in the organizational field. According to Morgan, metaphors are images that embody (or express) ideas about the organization as a whole, and about the people and the relationships within the workplace. He proposed distinguishing metaphors into “primary or root metaphors” and “second-order metaphors” (Morgan, 2016). The former are the well-known eight metaphors—machine, organism, brain, culture, political system, psychic prison, flow and transformation, and instrument of domination—described in the 1986 book, while the latter are possible other metaphors which the first eight may generate.

## The Metaphors of a Trade Union Organization

Taking our cue from the works of Morgan (2016) and Schoeneborn et al. (2016), the following pages describe the three primary metaphors (political system, organism, and culture) and four secondary metaphors (battleground, invisible partner, collective conviviality, and compass) which emerged from our analysis of the narratives provided by the managers who participated in the research.

### The Trade-Union as Battleground

“The union is like an army! Nothing has changed, the companies and their managers look after their own interests, and we have to be well-organized to protect the workers. Every day we have disputes that indicate that companies are not driven by ethical principles but merely by economic interest. We have to defend ourselves and defend the people!” (Metalworkers sector union manager, 30 years of service).

As can be seen from this excerpt, one secondary metaphor represents the union as an organization that can deploy the necessary resources to combat the predatory instincts of employers. The union is like an “army.” The relations between the two parties (one company and one trade-union) are characterized by the theme of confrontation, as if they were living in a continuous “battleground” situation. The keywords that qualify this sub-metaphor are: “army,” “defense,” “fight,” “abuse,” “exploitation,” “opposition,” “workers vs. bosses,” “factory,” “wage recovery,” “defending jobs,” and “the other side.”

These are all words that define the organization as a system based on sides in continuous conflict. To survive, you must “take sides” and face your enemy in a battle that will never end because it constitutes the organization itself. On a battlefield which is threatening and made even more dangerous by the market crisis, the only chance of survival is to think of oneself as a military organization with clear roles and a chain of command with which to organize a defense from (or an attack against) enemies.

This metaphor connects to the root metaphor of “the organization as a *political system*.” According to this image, the organization is conceived of as a system of government which contains interest groups, conflicts, negotiations, and power struggles. The first keyword that qualifies this concept is “exploitation,” which refers to an idea of organization as an instrument of exploitation in favor of élites and to the detriment of the workers. The second keyword is “domination;” this expresses the control of managers over workers forced to operate according to “times and methods” imposed upon them in order to make the ruling class rich. The third keyword of this root metaphor is “conflict,” which refers to the only type of relationship conceivable within this organizational field.

In this scenario, the ability to give value to intangible assets and in particular, to human and relational capital seems to be very limited. In fact, a simplified idea emerges of the organizational actor conceived of as a “soldier,” who has to serve one predetermined cause: to fight whatever the cost. The stakeholders are possible allies but within a non-negotiable “frame.” The roles seem to be crystallized and identified by the respective positions occupied. The heads of corporate companies are symbolized as “enemies,” and the stakeholders in the surrounding area are interlocutors with whom formal relations may be established arising from the institutional mandate. There is no possibility of imagining new creative relationships, a breeding ground for possible innovative ideas.

The representations of relational capital, too, are very limited. Little value is attributed to cultivating good internal relationships. Representation is only made available to a specific category of workers, namely those hired and employed permanently by the company. No other opportunities are envisaged, for example as regards the category of atypical workers, which is increasingly numerous nowadays. The union mainly identifies with one part of the workers and leaves aside the newly emerging job profiles.

### The Trade-Union as an Invisible Partner

“We offered our support to a small-to-medium-sized company to manage a restructuring process that involved the expulsion of many employees. Unfortunately, to save some jobs, we had to help the HR managers to do a dirty job, which at least saved the younger workers” (Electricity sector union manager, 22 years of service).

Unlike the previous one, the second metaphor brings into play the possibility of “making an alliance with the enemy” to externalize the threat of survival. What endangers the survival of the organization is the extreme competition which is typical of the neoliberal context, and it is in this scenario that the union presents itself to companies as an ally.

In this cluster, the metaphor which represents the trade union organization was proposed by a highly experienced interviewee who identified the trade union's role as the “invisible partner” who works on behalf of the management of the company. The trade unionists which we have included under this metaphor kept using the following words and expressions: “delocalization,” “economic-financial crisis,” “job insecurity,” “survival of the company,” and “partnership with the organization.” These terms clearly indicate the greatest challenges that companies have to face today: the delocalization of production, extreme levels of competitiveness, and the pressure to contain the cost of labor.

In order to face these challenges, the trade unionists in this cluster support a total identification with the aims of the employers, to the point of explaining to the workers, for example, the need for the restructuring plans, or making the organizational skills the trade union has honed available to the HR management of small businesses.

In our opinion, the metaphor of the “invisible partner” represents another new version of the root-metaphor of organization as “political system” mentioned above: in a “power struggle” situation, what is needed here are not clashes or battles, but a system of alliances with which to face a mutual enemy, constituted by opposing companies which are the competition. The interviewees who used this metaphor gave little importance to the contribution of union staff in supporting the union strategy.

The vision that emerges focuses on the ability of union managers to come to political/strategic agreements with the heads of the corporates in order to help them survive the market. The trade union organization is narrated as a hierarchical structure where all attention is to be devoted to sustaining the union's relational capital, by which is meant the ability to look after the relationship with stakeholders, industrial associations, company management, and strategic institutional partners. In this cluster, representation is understood in a way similar to how the first cluster understands it, but it is performed differently. The union has to represent the workers employed in the companies and safeguard their jobs.

It is a question of going along as much as possible with entrepreneurs so as to “support” the company's competitiveness in every way. This strategy makes it possible to represent workers' interests by turning the concept of representation upside down. By directly safeguarding the interests of employers, the workers are indirectly protected. This concept of “paradoxical” representation—I'm supporting the other side so as not to damage the workers too much—produces a very centralized vision of the organization. A few individuals decide the strategies, and then other people implement them.

### The Trade Union as a Vendor of Services

"The role of the union is to contribute to the survival of the organizations where it is active. The time for opposition is over. Now we all need to sit around a table and work out what can be done to help company management get people who are committed to working for the common good; we need to go easy on those pointless “68-style conflicts! We ourselves have to evolve as an organization to enable companies to change faster” (Chemical sector union manager, 25 years of service).

The third metaphor evoked places the emphasis on the importance of making the trade union financially stable by developing a clear positioning in the market. Here, the union managers are identifying themselves with an image proposed by one of the interviewees who compared the union to a multi-service organization that has to “sell its products.” In this scenario, the union becomes a “market” offering a range of different services in which it has acquired expertise. Here are some of the services mentioned: assisting members with income tax returns, offering social care, and organizing social tourism packages.

According to this metaphor, the organization is an *organism* which is able to evolve and adapting itself to changing market demands. From being the guarantor of the workers, the union has now become an employer. The managers in this cluster highlight the need for a change of “purpose,” in the name of survival. Organizations survive if they are capable of adapting to market needs and have a product they can sell. The recurring expressions we hear from these managers are: “flexibility,” “efficiency,” “autonomy,” “customer,” “products for customers,” “profitable services,” and “market visibility.” The organization is described as an entity which knows how to innovate and adapt in response to the challenges represented by the environment [read: the market]. In this conception, the trade union is a productive organization which in order to survive, has to place its goods and services on the market.

The interviewees present human capital as a strategic asset. The possibility to consolidate innovative services depends on the ability of the people who are part of the union to identify emerging product lines. Some examples of these are: leisure-time management services, assistance for pensioners, citizen tax consultancy services. People are asked to be innovators with regard to the products to be launched on the market. Relational capital is seen as an important, but not strategic asset. The word innovation is used above all in connection with the creative ability of those people who can come up with answers to new needs. Here, the concept of representation broadens almost to the point of becoming meaningless, because it encompasses every possible need the workers may have. The meaning of trade union action changes: the aim of defending the workers becomes broader and links up with the theme of social welfare protection.

### The Trade Union as a Convivium/Sextant

“The trade union is an organization that can create a new culture of coexistence in the workplace! There is the possibility to find new paradigms of social functioning at this point in our history” (Banking executive, 22 years of service).

In this cluster, we identified two metaphors, representing the image of conviviality and of the sextant, respectively. The first image is proposed in the work by Jermier and Forbes (2016), which draws inspiration from the classic *Tools for Conviviality* by Illich (1973). In this case, the emphasis is placed on the relationships that the union has built or should build: sincere, aimed at fostering an authentic bond between people, and not based on mutual exploitation (Illich, 1973). According to Illich, convivial effectiveness is possible through an organizational leadership not centered on technical and instrumental rationality.

The other sub-metaphor—that of the trade union conceived of as a sextant—captures the union's uncertainty in its ability to chart a credible course, both internally and in the outside world. In this cluster, the trade unionists interviewed seem to be wondering about the actual strength of the union's identity and values; the basic question seems to be linked to what types of relationships can be built in the workplace and what new position the union should occupy. The unionists in this cluster do not have a clear answer to the issues emerging. The questions remain open, and they support an attitude of actively seeking out a new identity that does not completely exclude the union's core values.

In this last cluster, the recurring terms are: “values,” “identity,” “research,” “the social value of the union,” “purpose.” The meaning of these terms, which evoke the trade union's search for a new identity, has led us to conclude that the root metaphor of this cluster is *culture*: this is an agricultural image being used to interpret organizations: just as in agriculture, a man takes care of plants and animals and helps them grow, in the same way, society and organizations devise models for their members' development which are mirrored in the systems of knowledge, ideologies, values, laws, rites and daily rituals. In literature, organizational culture is not interpreted as a fixed, unchangeable *thing*; it is a process which is based on a network of shared values and meanings, continuously (re)negotiated on the basis of daily *problem-defining* and *problem-solving* actions taken by the organizational actors.

In this position, the intangible capital is the source of the trade union association's renewal. Human capital is re-signified in terms of values capable of building a credible trade union identity within the present historical context. This involves creating spaces for discussion and exchange between people, in which the organizational processes support new forms of coexistence in the workplace.

Relational capital, too, is noticeably present in the narratives of these interviewees; here, there is an awareness that the process of seeking out and determining the new identity cannot rely only on personal and internal resources, because it requires the participation of all those stakeholders who have expectations of the union.

Representation, too, takes on new forms and arises from a dialogue between the subjects, without being connected to any particular category of workers (such as employees or people expelled from the world of work). It embraces the broad concept of defense of civil co-existence in the workplace. The union does represent a group: it represents those ideals of inclusion and integration necessary for civil co-existence.

## Conclusions

This study describes how, when faced with their own internal crisis, the managers of a trade union organization develop different new frames with which to rethink its purpose and possible actions, and the role of the organization's intangible assets. In fact, through this work of collective action framing, the union is trying to rediscover its origins as a social movement and rebuild its capacity to mobilize workers through renewed organizing and associated initiatives (Kelly, 2012). In this process, we argue that in order to guarantee their future, unions must reinforce their internal strengths, and their intangible assets in particular.

In the metaphor of the battleground and the invisible partnership, the crisis of the union is attributed to the demands coming from an increasingly turbulent external environment, and to the social and work inequalities associated with the neo-liberal model. In this scenario, the union managers see the union's main purpose as fighting to continue to defending the survival of the workers (and of the company).

In this “struggle,” the value attributed to intangible assets is very limited. The workers are perceived in terms of the “categories” and “roles” covered; their contribution would seem to be limited to the force they are able to exert in an open war/battle with the ruling class.

Internal relations are represented as the construction of alliances, on the one hand, or as hostile moves against the enemy, on the other. The image of the organization is reduced to its hierarchies, in particular by restricting the organizational gaze to the managerial level and the “grassroots.”

While on the one hand, the *amicus–hostis* dichotomy would appear to be a possible strategy with which to mobilize trade unionists to meet the need for renewal, it does seem to present some pitfalls. These include: the impossibility of representing the complexity of the organizational actors and stakeholders; the difficulty of starting a dialogue among all the organizational actors; the risk of isolating oneself and losing contact with the demands and needs of the new categories of workers.

Faced with the problem of survival and how to adapt to the requirements of the market, the metaphor of “supplier of services” is a new way of repacking the purpose of the trade union. From being the defender of the workers' interests, the union now moves on to become a “proactive producer” of services, in direct response to the workers' needs.

Here, the intangible assets, and human capital in particular, are perceived as being more important: people's knowledge and skills become fundamental in order to imagine new products and to make them available. However, the risk of this perspective seems to be that of a weakening of the true purpose of the union, which might be confused with any service organization.

Intangible assets are also attributed value, but only in a partial sense: the relationships between organizational actors, potential new members and stakeholders are imagined as mere commercial/economic transactions (supply-demand exchange), while in the background lies the idea of participation and dialogue in the co-planning of the service.

In the metaphor of the convivium, managers present the purpose of the new union as an open option, with respect to which members are mobilized to get involved in co-planning the future of their organization.

In this perspective, value is attributed in particular to both human and relational capital: the union's members, but also the stakeholders, are represented as “authors” of the union's new identity; the relationships between all the individuals involved are seen as resources for research, discussion, and dialogue about future perspectives.

The mobilization of trade unionists concerns precisely being involved in a process of research and planning of a new form of social relations and solidarity between workers; a process that is still ongoing but based squarely on human capital.

The consequences of these different positions vary. From the point of view of how to manage change, in accordance with Benevene et al. (2017), this study highlights the importance for trade union managers to develop a greater awareness of the intangible asset paradigm, thereby obtaining a clearer idea of their organization's potential for growth. Poor representation and limited use of the power of intangible assets prevents organizations and their managers from exploiting all their benefits.

The implications of this study for the HR function are that managers should be stimulated to rethink their management styles, and it highlights how varying approaches to people management in trade union organizations (made evident here by metaphors) can make a big difference to the value attributed to intangible assets. The metaphors point to four ways of conceiving the role of people in the organization. Each of these four ways attributes a different value to intangible assets. The fourth metaphor conceives of people as an asset to the organization, as the bearers of ideas and knowledge that can contribute to the organization's success. On reading this paper, managers might be persuaded to reflect on how to better exploit the intangible assets in their organization by creating work environments that enable people to have ever-greater degrees of participation.

Our study highlights one of the possible routes that the union should follow to find a credible identity. In fact, we have described the role played by the metaphors used by the organizational actors when they ask themselves what their organization consists of in terms of intangible assets (Marr et al., 2003). Being aware of the extent of one's intangible assets makes it possible to implement the process of organizational repositioning and stakeholder mobilization and makes this process much easier. The more that managers are aware of the organization's intellectual and relational capital, the easier it is to activate these precious forms of capital, make them visible, and use them to sustain a process of transition.

## Data Availability Statement

The raw data supporting the conclusions of this article will be made available by the authors, without undue reservation.

## Ethics Statement

Ethical review and approval was not required for the study on human participants in accordance with the local legislation and institutional requirements. Written informed consent for participation was not required for this study in accordance with the national legislation and the institutional requirements.

## Author Contributions

SR, AB, and LG conceived of the idea. LG developed the theoretical framework. AB took care of the methodological section and the conclusions. SP contributed to the article review and its reference section. SR wrote the data analysis and discussion sections. All authors contributed to the article and approved the submitted version.

## Conflict of Interest

The authors declare that the research was conducted in the absence of any commercial or financial relationships that could be construed as a potential conflict of interest.
